# Risk factor prediction of severe postoperative acute kidney injury at stage 3 in patients with acute type A aortic dissection using thromboelastography

**DOI:** 10.3389/fcvm.2023.1109620

**Published:** 2023-02-09

**Authors:** Xin-Liang Guan, Lei Li, Hai-Yang Li, Ming Gong, Hong-Jia Zhang, Xiao-Long Wang

**Affiliations:** Department of Cardiac Surgery, Beijing Aortic Disease Center, Beijing Anzhen Hospital, Capital Medical University, Beijing Institute of Heart, Lung, and Blood Vessel Diseases, Beijing Laboratory for Cardiovascular Precision Medicine, Beijing Engineering Research Center of Vascular Prostheses, Beijing, China

**Keywords:** acute type A aortic dissection, acute kidney injury, thromboelastography (TEG), risk factor, continuous renal replacement therapy (CRRT)

## Abstract

**Objective:**

Perioperative blood transfusions and postoperative drainage volume not only are the commonly recognized risk factors for acute kidney injury (AKI) but also are indirect indicators of coagulopathy in patients with acute type A aortic dissection (ATAAD). However, standard laboratory tests fail to accurately reflect and assess the overall coagulopathy profile in patients with ATAAD. Thus, this study aimed to explore the association between the hemostatic system and severe postoperative AKI (stage 3) in patients with ATAAD using thromboelastography (TEG).

**Methods:**

We selected 106 consecutive patients with ATAAD who underwent emergency aortic surgery at Beijing Anzhen Hospital. All participants were categorized into the stage 3 and non-stage 3 groups. The hemostatic system was evaluated using routine laboratory tests and TEG preoperatively. We undertook univariate and multivariate stepwise logistic regression analyses to determine the potential risk factors for severe postoperative AKI (stage 3), with a special investigation on the association between hemostatic system biomarkers and severe postoperative AKI (stage 3). The receiver operating characteristic (ROC) curves were generated to assess the predictive ability of hemostatic system biomarkers for severe postoperative AKI (stage 3).

**Results:**

A total of 25 (23.6%) patients developed severe postoperative AKI (stage 3), including 21 patients (19.8%) who required continuous renal replacement therapy (RRT). Multivariate logistic regression analysis demonstrated that the preoperative fibrinogen level (OR, 2.02; 95% CI, 1.03 to 3.00; *p* = 0.04), platelet function (MA level) (OR, 1.23; 95% CI, 1.09 to 1.39; *p* = 0.001), and cardiopulmonary bypass (CPB) time (OR, 1.01; 95% CI, 1.00 to 1.02; *p* = 0.02) were independently associated with severe postoperative AKI (stage 3). The cutoff values of preoperative fibrinogen and platelet function (MA level) for predicting severe postoperative AKI (stage 3) were determined to be 2.56 g/L and 60.7 mm in the ROC curve [area under the curve (AUC): 0.824 and 0.829; *p* < 0.001].

**Conclusions:**

The preoperative fibrinogen level and platelet function (measured by the MA level) were identified as potential predictive factors for developing severe postoperative AKI (stage 3) in patients with ATAAD. Thromboelastography could be considered a potentially valuable tool for real-time monitoring and rapid assessment of the hemostatic system to improve postoperative outcomes in patients.

## Introduction

Acute kidney injury (AKI) has become a frequent and serious complication characterized by staggeringly high morbidity and mortality in patients with acute type A aortic dissection (ATAAD) after total arch replacement (TAR) combined with a frozen elephant trunk (FET) implant (1–3). Unlike in other cardiovascular surgeries, the incidence of AKI after thoracic aortic surgery is higher and varies considerably (4–7). However, some studies demonstrated that only patients with severe postoperative AKI (stage 3) had lower long-term survival—not patients with postoperative AKI (stages 1 and 2) (1, 2). Therefore, early identification and prompt prevention of potential risk factors for severe postoperative AKI (stage 3) play an important role in improving the overall prognosis of patients with ATAAD.

The majority of experts (3, 5) believe that excessive perioperative bleeding, blood transfusion, or postoperative drainage volume are currently identified as independent relevant risk factors for ATAAD-AKI. To some extent, the relationship between the hemostatic system (bleeding, transfusion, and drainage) and ATAAD-AKI has already been discussed in previous studies (8–10). However, there is a lack of uniform data regarding the association between hemostatic system biomarkers and severe postoperative AKI (stage 3) in patients with ATAAD. As it provided information not only about the dynamics of clot formation and clotting factors but also about the function of platelet and fibrinogen, thromboelastography (TEG) has been described as a prospective tool in patients undergoing non-complex cardiac surgery (11). Nevertheless, only a few studies have investigated the dynamics of the hemostatic system using TEG in the acute and complex settings of aortic dissection. Thus, the purpose of our study was to explore the incidence and risk factors for severe postoperative AKI (stage 3) among patients with ATAAD after emergency aortic surgery, with special emphasis on the relationship between the hemostatic system and the severity of postoperative AKI.

## Material and methods

### Study design

In this single-center prospective study, we analyzed the association between the hemostatic system and severe postoperative AKI (stage 3) in 106 patients with ATAAD who underwent aortic arch surgery using the preoperative routine laboratory test results and TEG analysis at Beijing Anzhen Hospital, Capital Medical University, China. The plasma fibrinogen concentration was tested using the Clauss method. A single team performed all procedures. The protocol of this study was approved by Anzhen Hospital's Ethics Committee (No. 2018004), and consent was obtained from the patients or their relatives.

### Patient population

**Figure 1:**
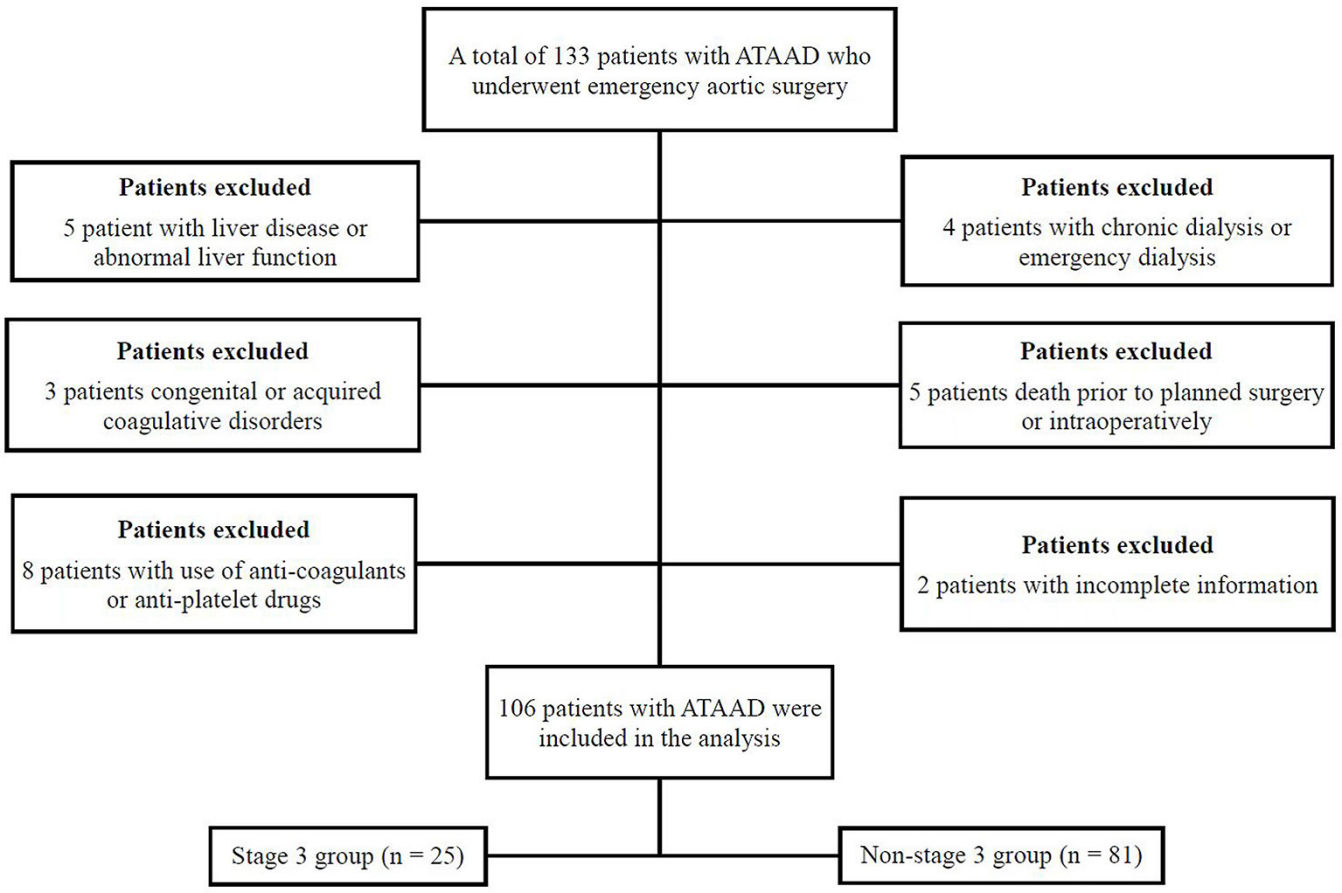
A flowchart of the study cohort.

From June 2020 to December 2021, a total of 106 patients with ATAAD per the Stanford classification were eligible for inclusion in the study at the Institute of Cardiac Surgery, Beijing Anzhen Hospital, Capital Medical University, China (Figure 1). All emergency TAR combined with a FET implant with cardiopulmonary bypass (CPB) involving moderate hypothermic circulatory arrest (HCA) with or without aortic valve operations were collected for further analysis. Patients were recruited consecutively on the condition that they agreed to provide informed consent. The exclusion criteria included congenital or acquired coagulative disorders, liver disease or abnormal liver function, preoperative use of anti-coagulants or antiplatelet drugs, death before planned surgery, preoperative chronic dialysis within the past month or emergency dialysis before surgery, and incomplete clinical data.

### Measurements and variable definitions

The diagnosis of postoperative AKI stage 3 was based on the Kidney Disease: Improving Global Outcomes (KDIGO) criteria (12): a threefold increase or more above baseline or an increase in serum creatinine (sCr) to ≥ 4.0 mg/dl (≥ 353.6 mmol/l) or the initiation of renal replacement therapy (RRT) or, in patients under 18 years of age, a decrease in estimated glomerular filtration rate (eGFR) to <35 ml/min per 1.73 m^2^ (Table 1). The diagnosis of ATAAD was confirmed by a contrast-enhanced computed tomography (CT) scan, with the onset of symptoms onset within 48 h. Intraoperative bleeding was defined as blood loss that was collected and quantified using intraoperative cell salvage and surgical gauze swabs. Continuous RRT was defined as the need for continuous hemofiltration or hemodialysis after surgery.

**Table 1:** KDIGO stages of AKI according to sCr levels and urine output.

**Stage**	**sCr**	**Urine output**
1	1.5–1.9 times baseline or ≥ 0.3 mg/dL (≥26.5 mmol/l) increase	<0.5 ml/kg/h for 6-12 h
2	2.0–2.9 times baseline	<0.5 ml/kg/h for ≥12 h
3	> 3.0 times baseline or increase in sCr to ≥ 4.0 mg/dl (≥353.6 mmol/l) or initiation of RRT or in patients <18 years, decrease in eGFR to <35 ml/min per 1.73 m^2^	<0.3 ml/kg/h for ≥24 h or anuria for ≥12 h

AKI, acute kidney injury; Egfr, estimated glomerular filtration rate; KDIGO, Kidney Disease: Improving Global Outcomes; sCr, serum creatinine.

### TEG analysis

After taking blood samples in the emergency department, the samples were immediately transferred to a clinical laboratory in our hospital. According to the manufacturer's instructions, professional staff performed TEG analysis using the TEG 5000 analyzer (Haemoscope, Niles, IL). The following EG parameters were tested: R time is the period from the initiation of the test to the initial fibrin formation (representing thrombus formation initiation). K time is the period from the beginning of clot formation until the amplitude of the curves reaches 20 mm (representing the dynamics of clot formation). Maximum amplitude (MA) is a direct measure of the highest point on the TEG curve (which represents platelet function). Theαangle is the angle between the line in the middle of the TEG tracing and tangential to the body of the TEG tracing (represents the kinetics of fibrin buildup and cross-linking).

### Surgical procedures

Under standard anesthetic management, emergency aortic surgery refers to TAR using a tetra-furcate vascular graft in combination with the implantation of FET into the descending aorta under moderate HCA. Briefly, the procedure involved cannulation of the right axillary artery and right atrium for CPB and selective antegrade cerebral perfusion [5–15 mL/(kg·min)] at a nasopharyngeal temperature of approximately 26–28°C. After systemic heparinization, the proximal aortic root operation was carried out based on the lesions of the aortic root during the cooling period. The sequence of aortic arch reconstruction was proximal descending aorta, left carotid artery, ascending aorta, left subclavian artery, and innominate artery. After the distal anastomosis was completed, CPB was restarted, and the patient was gradually rewarmed to a normal temperature. Other concomitant operations were conducted during the rewarming period. Upon completion of the repair and adequate rewarming, the patient was extubated from CBP.

### Statistical analysis

The normality of the data distribution was tested using the Kolmogorov–Smirnov test. Continuous data with a normal distribution were expressed as mean ± standard deviation (SD), and continuous data with a non-normal distribution were expressed as median (25 and 75^th^ percentile); categorical variables were expressed as *n* (%). For comparison, independent sample *t*-tests or the Wilcoxon rank sum tests were analyzed for continuous variables. The chi-squared test or Fisher's exact test was used for categorical variables. The univariate logistic regression analysis was used to compare baseline characteristics between two groups for severe postoperative ATAAD-AKI (stage 3), and the multivariate stepwise logistic regression model was carried out to identify possible risk factors (*p* < 0.01) for severe postoperative ATAAD-AKI (stage 3). The receiver operating characteristic (ROC) curve was performed to further evaluate the predictive ability of risk factors for severe postoperative ATAAD-AKI (stage 3). For all analyses, a two-tailed value of p of < 0.05 was considered statistically significant. All statistical analyses were conducted using SPSS 18.0 (SPSS, Inc., Chicago, IL).

## Results

### Incidence of postoperative ATAAD-AKI

Based on the KDIGO criteria, our study's incidence of postoperative ATAAD-AKI was 53.8% (57/106). Among them, the prevalence of severe ATAAD-AKI was 38.6% for stage 1 (22 cases), 17.5% for stage 2 (10 cases), and 43.9% for stage 3 (25 cases). In total, 21 patients (19.8%) needed continuous RRT after the operation. Renal malperfusion occurred in 14 patients (13.2%) preoperatively. Among them, four (28.6%) patients developed postoperative AKI. The mean age of patients with AKI was 48.2 ± 10.5 years, and the data involved 42 men and 15 women in the AKI group. Chest pain (94.4%) represents one of the most frequent symptoms in patients with ATAAD. Hypertension was confirmed in 84 out of the 106 patients. Only 6.6% of patients have Marfan syndrome. With regard to imaging data, the clot-filled false lumen appeared on enhanced CT in 65 patients. In addition, 79 patients suffered dissections extending below the diaphragm, and the remaining 27 patients had dissections terminating above the diaphragm (Table 2).

**Table 2:** Characteristics of the study patients with ATAAD at baseline.

**Characteristics**	**Stage 3 (n = 25)**	**Non-stage 3 (n = 81)**	***p*** **-value**
**Demographic data**			
Age, year	50.2 ± 9.4	47.5 ± 10.8	0.26
Male, %	15 (60.0)	64 (79.0)	0.11
BMI, kg/m^2^	27.3 ± 3.4	25.9 ± 3.9	0.11
**Medical history**			
Hypertension, %	23 (92.0)	61 (75.3)	0.07
Diabetes mellitus, %	1 (4.0)	6 (7.4)	0.55
Cerebrovascular disease, %	1 (4.0)	4 (4.9)	0.85
Coronary artery disease, %	0	6 (7.4)	0.17
Smoking history, %	14 (56.0)	35 (43.2)	0.26
Drinking history, %	9 (36.0)	14 (17.3)	0.45
Marfan syndrome, %	4 (16.0)	3 (3.7)	0.03
**Preoperative condition**			
Alanine amino transaminase, U/L	24.9 ± 5.8	33.4 ± 5.5	0.18
sCr, umol/L	94.5 ± 32.8	84.3 ± 27.9	0.13
eGFR, mL/(min·1.73m^2^)	85.8 ± 12.4	84.7 ± 13.2	0.36
White blood cells, × 10^3^/mm^3^	12.4 ± 3.7	11.0 ± 3.7	0.11
Neutrophil ratio, %	84.4 ± 5.1	77.9 ± 9.3	0.001
Hemoglobin, g/dL	132.1 ± 14	137.0 ± 16.9	0.19
Platelet counts, × 10^3^/mm^3^	141.8 ± 40.7	185.0 ± 76.7	0.008
Fibrinogen level, g/L	2.3 ± 1.1	3.9 ± 1.6	< 0.001
FDP, ug/mL	35.8 (18.7, 65.2)	11.7 (6.5, 27.4)	< 0.001
D-Dimer, ng/mL	2378 (1918, 7726)	1085 (591, 2526)	0.001
LVEF, %	64.7 ± 5.3	62.0 ± 6.0	0.05
Aortic root size, mm	40.8 ± 7.3	41.0 ± 8.3	0.94
Ascend aorta size, mm	46.6 ± 7.1	45.4 ± 7.6	0.50
Aortic regurgitation, %	11 (44.0)	36 (44.4)	0.97
Renal malperfusion, %	4 (16.0)	10 (12.3)	0.64
**TEG**			
R time (min)	5.5 ± 1.6	5.9 ± 3.1	0.48
K time (min)	2.0 ± 0.9	1.7 ± 1.1	0.35
MA (mm)	55.7 ± 7.7	65.1 ± 6.8	< 0.001
α angle (degree)	63.6 ± 7.3	67.1 ± 9.3	0.08
**Operation details**			
Bentall+TAR+FET, %	12 (48.0)	28 (34.6)	0.23
Combined with CABG, %	2 (8.0)	6 (7.4)	0.92
The duration of operation, hour	9.35 ± 2.0	8.1 ± 1.8	0.004
CPB time, min	242.0 ± 69.9	202.2 ± 49.0	0.002
Aortic cross clamp time, min	135.8 ± 39.8	120.9 ± 43.8	0.13
The duration of HCA, min	28.8 ± 7.0	26.5 ± 9.4	0.23
Nasopharyngeal temperature, °C	22.3 ± 1.4	23.2 ± 2.0	0.06
Rectal temperature, °C	25.1 ± 2.0	24.5 ± 2.4	0.45
Intraoperative blood loss, mL	1592 ± 615	1462 ± 767	0.44
Intraoperative amount of plasma, mL	500 (0, 1000)	400 (100, 600)	0.25
Intraoperative amount of RBC, mL	600 (0, 750)	300 (0, 600)	0.04
24 h postoperative drainage	600 (380, 910)	650 (500, 925)	0.58
48 h postoperative drainage	1000 (725, 1195)	1050 (720, 1550)	0.49
**Postoperative outcomes**			
In-hospital mortality, %	4 (16.0)	4 (4.9)	0.07
Length of hospital, day	15 (12, 22)	14 (10, 17)	0.16
Length of ICU, day	8.0 (6, 12)	2 (1, 4)	< 0.001
Continuous RRT, %	21 (84.0)	0	< 0.001
Severe hypoxemia, %	18 (72.0)	21 (25.9)	< 0.001
Reoperation for bleeding, %	3 (12.0)	5 (6.2)	0.34
Low cardiac output syndrome, %	2 (8.0)	4 (4.9)	0.56
Cerebral infarction or bleeding, %	2 (8.0)	7 (8.6)	0.75
Multi-organ failure, %	7 (28.0)	4 (4.9)	0.004
Sepsis, %	7 (28.0)	9 (11.1)	0.04

Results are expressed as n (%), mean ± SD or median (interquartile range). AKI, acute kidney injury; ATAAD, acute type A aortic dissection; BMI, body mass index; CABG, coronary artery bypass grafting; CPB, cardiopulmonary bypass; eGFR, estimated glomerular filtration rate; FET, frozen elephant trunk; FDP, fibrinogen degradation products; HCA, hypothermic circulatory arrest; ICU, intensive care unit; LVEF, left ventricular ejection fraction; MA, maximum amplitude; RBC, red blood cell; RRT, renal replacement therapy; sCr, serum creatinine; TAR, total arch replacement; TEG, Thromboelastography.

### Baseline characteristics

Based on the KDIGO criteria, the patient population was divided into two groups: the stage 3 group and the non-stage 3 group. Patient baseline demographic information is presented in Table 2. Marfan syndrome was more common in the severe postoperative AKI (stage 3) group (16.0 vs. 3.7%, *p* = 0.03) according to the medical history. The preoperative routine laboratory tests between the two groups are also summarized in Table 2. The neutrophil ratio, fibrinogen degradation products (FDP), and D-dimer were higher in the severe postoperative AKI (stage 3) group compared to patients in the non-stage 3 group (*p* = 0.001, *p* < 0.001, and *p* = 0.001, respectively). Nevertheless, platelet counts and fibrinogen levels were relatively lower in the severe postoperative AKI (stage 3) group (*p* = 0.008 and *p* < 0.001, respectively). In addition, TEG parameters showed that the MA level (platelet function) was lower in the severe postoperative AKI (stage 3) group when compared to the non-AKI stage 3 group (*p* < 0.001). Other TEG parameters, such as R time, K time, andαangle, did not differ significantly between the two groups.

### Surgical characteristics and postoperative outcomes

Patient surgical details are shown in Table 2. Our data demonstrated that operation time and CPB time were all prolonged in patients with severe postoperative AKI (stage 3) (*p* = 0.004 and *p* = 0.002). Regarding nasopharyngeal or rectal temperature, no significant differences were observed between the two groups. Notably, there was a higher intraoperative amount of red blood cells (RBC) in the severe postoperative AKI (stage 3) group than in the non-stage 3 groups (*p* = 0.04). Although there was a similarity with respect to in-hospital mortality between the two groups (*p* = 0.07), the postoperative complications were indeed more serious and complicated in patients with severe postoperative AKI (stage 3), such as a longer intensive care unit (ICU) stay, continuous RRT, severe hypoxemia, multi-organ failure, and sepsis (*p* < 0.001, *p* < 0.001, *p* < 0.001, *p* = 0.004 and *p* = 0.04, respectively).

### Changes in preoperative MA level (platelet function) and fibrinogen level among AKI groups according to the AKI stages

**Figure 2:**
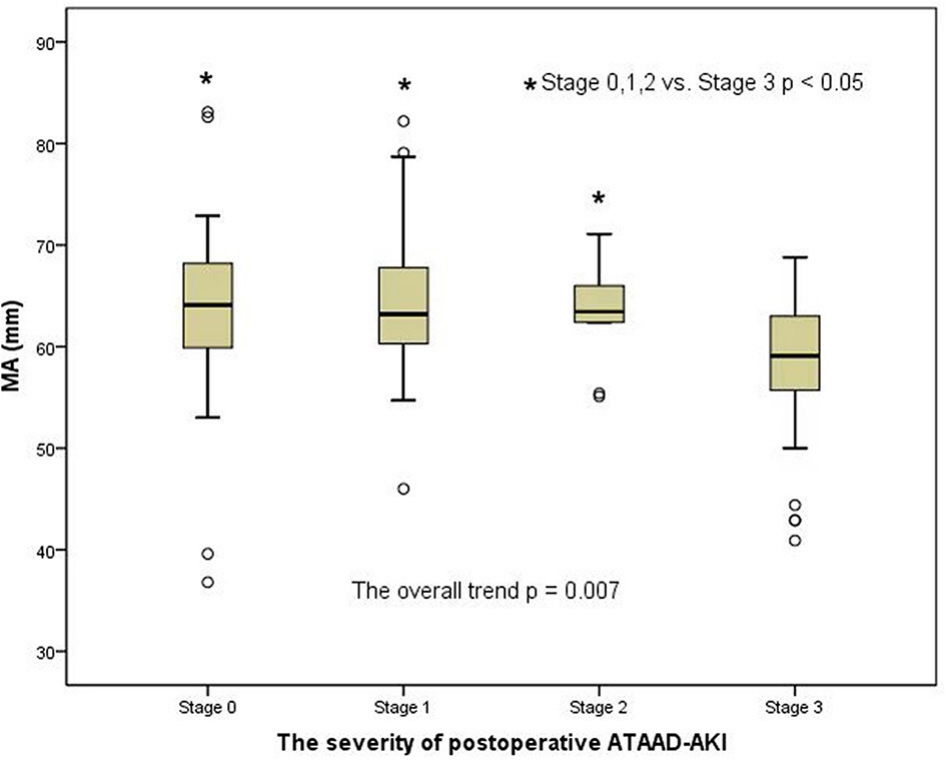
Changes in preoperative MA level (platelet function) among the AKI groups.

**Figure 3:**
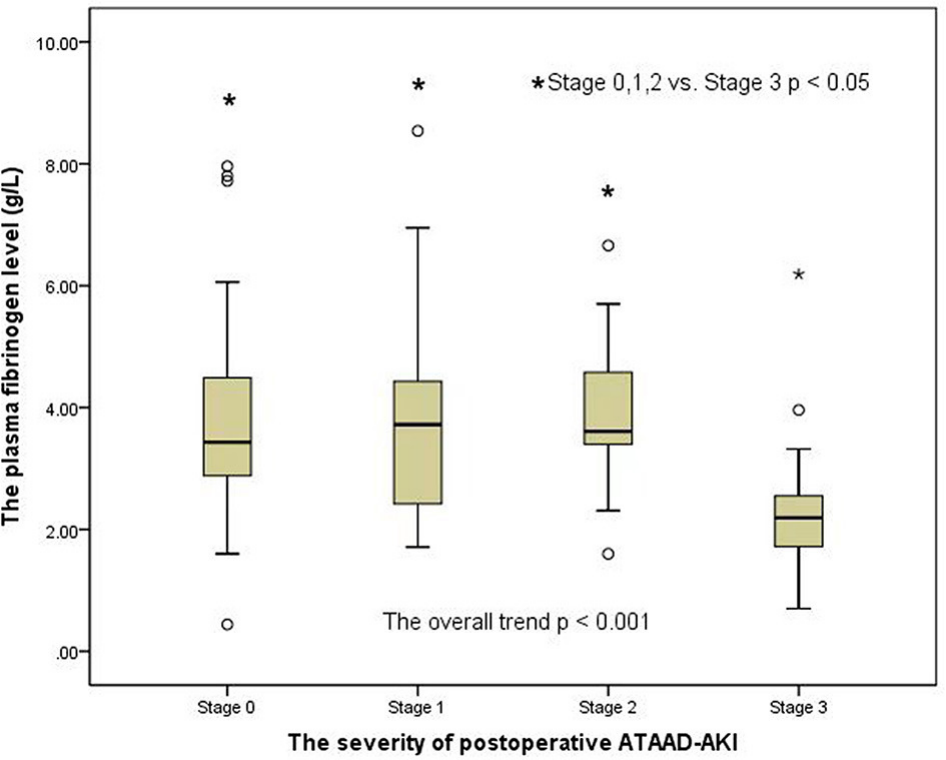
Changes in preoperative fibrinogen level among the AKI groups.

A clear distinction was observed in the preoperative MA level (platelet function) among the groups of patients with AKI when analyzed by the AKI stages in a general trend analysis (*p* = 0.007) (Figure 2). Similar to the overall analysis, the MA level (platelet function) was lower in postoperative AKI stage 3 compared with the stage 0, stage 1, and stage 2 groups (*p* = 0.001, *p* = 0.004, and *p* = 0.041, respectively). Moreover, there was also a significant distinction in preoperative fibrinogen levels among AKI groups in the overall trend analysis (*p* < 0.001) (Figure 3). Similarly, the preoperative fibrinogen level was lower in postoperative AKI stage 3 compared with other AKI stages (*p* < 0.001, *p* = 0.001, and *p* = 0.006, respectively).

### Univariate and multivariate logistic regression analysis associated with independent risk factors for severe postoperative AKI (stage 3)

The preoperative characteristics, such as neutrophil ratio, platelet counts, fibrinogen level, FDP, D-Dimer, and MA level (platelet function), were associated with related risk factors for severe postoperative AKI (stage 3) in the univariate analysis (*p* < 0.01). Furthermore, the duration of the operation and CPB time in operative variables might be linked to the risk of severe postoperative AKI (stage 3) in univariate logistic regression analysis (*p* < 0.01). To address issues of collinearity, a multivariate stepwise logistic regression analysis was conducted to identify the risk factors of severe postoperative AKI (stage 3), and the results are summarized in Table 3. Among the potential risk factors determined by a univariate analysis (*p* < 0.01), independent risk factors for severe postoperative AKI (stage 3) in patients with ATAAD were the preoperative fibrinogen level [OR, 2.02; 95% confidence interval (CI), 1.03 to 3.00; *p* = 0.04], the MA level (platelet function) (OR, 1.23; 95% CI, 1.09 to 1.39; *p* = 0.001), and longer CPB time (OR, 1.01; 95% CI, 1.00 to 1.02; *p* = 0.02) in multivariate logistic regression analysis.

**Table 3:** Risk factors for postoperative AKI (stage 3) in multivariate logistic regression analysis in patients with ATAAD.

**Risk factors**	**OR**	**95% CI**	***p*** **-value**
Neutrophil ratio, %	1.07	0.94–1.21	0.32
Platelet counts, × 10^3^/mm^3^	0.99	0.97–1.00	0.11
Low fibrinogen level, g/L	2.02	1.03–3.00	0.04
FDP, ug/mL	0.98	0.93–1.03	0.34
D-Dimer, ng/mL	1.01	0.98–1.04	0.33
Low MA (mm)	1.23	1.09–1.39	0.001
The duration of operation, hour	1.01	0.99–1.02	0.97
CPB time, min	1.01	1.00–1.02	0.02

AKI, acute kidney injury; ATAAD, acute type A aortic dissection; CI, confidence interval; CPB, cardiopulmonary bypass; FDP, fibrinogen degradation products; MA, maximum amplitude; OR, odds ratio.

### Predictive ability of a risk factor for severe postoperative AKI (stage 3)

**Figure 4:**
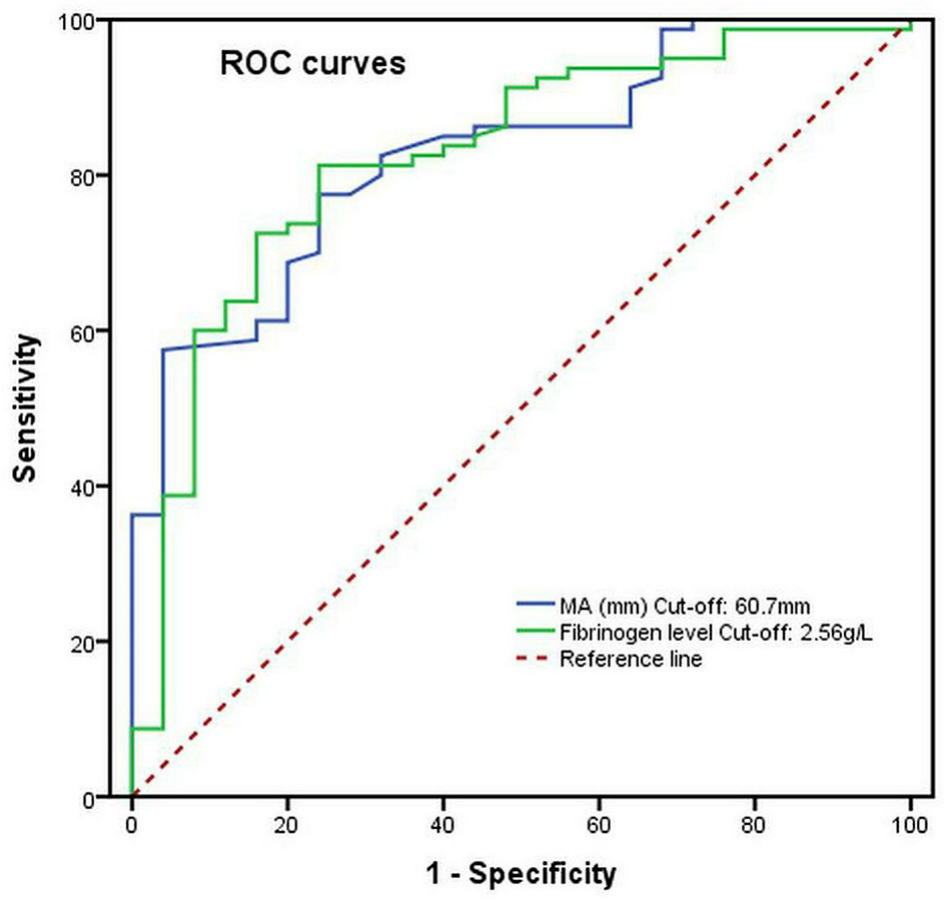
The preoperative MA level (platelet function) and fibrinogen level as predictive values of risk factors for severe postoperative AKI (stage 3) in patients with ATAAD by the ROC curve analysis.

The ROC curves were generated to explore the predictive ability and the cutoff value of risk factors for severe postoperative AKI (stage 3). As shown in Figure 4, the area under the curve (AUC) of the preoperative fibrinogen level and the MA level (platelet function) used for predicting severe postoperative AKI (stage 3) in patients with ATAAD were 0.824 (cutoff, 2.56 g/L; sensitivity, 81.3%; specificity, 76.0%; *p* < 0.001) and 0.829 (cutoff, 60.7 mm; sensitivity, 77.5%; specificity, 72.0%; *p* < 0.001), respectively.

## Discussion

The key conclusion of this study was that the preoperative fibrinogen and MA levels (platelet function) were independent predictive indicators for risk factors associated with severe postoperative AKI (stage 3) in patients with ATAAD after TAR combined with a FET, and it emphasized the association between hemostatic system biomarkers and severe postoperative AKI (stage 3). To the best of our knowledge, this is the first research to investigate the relationship between hemostatic system biomarkers and the severity of postoperative AKI in patients with ATAAD using TEG. With the aid of TEG parameters, early monitoring and identification of critically ill patients may be achieved for renal preventive and protective strategies.

The incidence of severe postoperative ATAAD-AKI (stage 3) in our study is 23.6% (25/106), and the required continuous RRT is 19.8% (21/106). A recently published investigation from Wang et al. (9) revealed that 23.8% of patients developed severe postoperative ATAAD-AKI (stage 3) after ATAAD surgery, including 16.6% of patients who received continuous RRT. Similarly, Chen et al. (7) also demonstrated that 47.9% of patients developed severe postoperative AKI (AKI stages 2 or 3) and 14.6% required continuous RRT. However, Ko (2) suggested that the incidence of developing severe postoperative AKI (stage 3) after aortic arch surgery was only 14%, and the rate of patients who needed continuous RRT was as low as 9%. The reason for these lower rates might be mainly attributed to the exclusion of emergency ATAAD surgery from that study. Due to the life-threatening aortic syndrome and the complexity of the urgent operation, it is unsurprising that the rates of severe postoperative AKI (AKI stage 3) and continuous RRT were up to 20% in our study. Increasing evidence suggests that increasing AKI severity is associated with an increase in mortality. Thus, TEG might be a suitable tool for early AKI diagnosis and the prediction of the need for RRT in patients with ATAAD.

The high mortality rate for emergency aortic surgery is challenging and is associated with high rates of perioperative bleeding and blood product transfusions (13, 14). Excessive perioperative bleeding and blood product transfusions represent one of the most common and feared complications in emergency aortic surgery. Massive blood transfusions are also considered an indirect marker of hemorrhage and a known risk factor for ATAAD-AKI (8, 10). Growing evidence (2, 3, 15) indicates that perioperative transfusions of large amounts of RBC and plasma were designed to determine independent risk factors for AKI. In line with these studies, the intraoperative quantity of RBC was higher in our study's severe postoperative AKI (stage 3) group. In addition, patients with major bleeding on preoperative dual antiplatelet therapy had more postoperative AKI, which indirectly showed the relationship between bleeding and AKI (16).

Wang et al. (9) showed that the logistic regression model identified the 24-h drainage volume after an aorta repair operation as another independent risk factor for postoperative AKI stage 3. Additionally, the multivariate logistic regression analysis similarly revealed that 72-h drainage volume was an important predictor of postoperative ATAAD-AKI in an overweight patient with ATAAD (6). Excessive drainage volume can disrupt homeostasis, induce pro-inflammatory states, and increase oxidative stress, which will contribute to the pathogenesis of AKI (17). Therefore, decreasing postoperative drainage volume is considered essential and may reduce the incidence of postoperative AKI. Although the association between drainage volume and ATAAD-AKI was not directly established in our research, it appears that excessive bleeding and transfusion did not provide any benefits for patients with ATAAD.

HCA- and CPB-induced coagulopathy in aortic surgery is already a well-accepted clinical pathological condition (18–20). The impairment of the hemostatic system is already caused by the contact of blood flow with the non-endothelialized walls of the false lumen before emergency aortic surgery (19, 21). In light of the activation of the hemostatic system preoperatively, our study primarily described preoperative changes in the hemostatic system in patients with ATAAD. Similar to some previous studies (21, 22), our study's routine laboratory tests and TEG documented fibrinogen, platelet, and clotting factor consumption and, ultimately, coagulopathy in the early preoperative period of ATAAD. To date, no similar study has been performed in which data on the association between TEG parameters of changes in the hemostatic system and severe postoperative AKI (stage 3) were obtained.

It is widely known that platelets and fibrinogen are critical for clot formation and clot strength. Therefore, increasing emphasis has focused on the importance of platelets and fibrinogen in reducing blood loss and improving prognosis (19). Notably, many guidelines (23–25) have recommended the use of fibrinogen concentrate and platelets to correct early coagulopathy. Some previous studies (19, 21, 26) consistently reported that fibrinogen is directly responsible for clot strength and can compensate for platelet function. Indeed, the shortage of platelets and fibrinogen might lead to perioperative bleeding and blood product transfusions associated with postoperative AKI. Our research confirmed that the multivariate logistic regression analysis identified the preoperative fibrinogen level and the MA level (platelet function) as independent risk factors for severe postoperative AKI (stage 3) in patients with ATAAD. Thus, we have reasons to believe that there are predictable and quantifiable changes in TEG parameters for severe postoperative AKI (stage 3) in ATAAD.

Because of the interaction between a non-pulsatile flow and the activation of an inflammatory response, a few studies (2, 8, 27, 28) had already confirmed that CPB is associated with increased postoperative AKI. Moreover, Englberger et al. (29) discovered that every additional 10-min increase in CPB time would lead to a higher risk of postoperative ATAAD-AKI. Similarly, Wang et al. (9) also illustrated that prolonged CPB duration is an independent risk factor for developing severe postoperative ATAAD-AKI (stage 3). In contrast, Kim et al. (3) and Li et al. (5) discovered that HCA time is associated with a risk for ATAAD-AKI in multivariable analysis but not CPB. Amano et al. (30, 31) also concluded that the duration of HCA was recognized as a surgical risk factor for postoperative ATAAD-AKI. However, Roh et al. (8, 29, 32) did not find a relationship between HCA time and ATAAD-AKI. We believe that, despite the inconsistency of those conclusions, there was no doubt that renal medullary ischemia or reperfusion injury induced by CPB or HCA might be the most important pathophysiological change associated with ATAAD-AKI.

Early identification and management of the hemostatic system might be lifesaving (33). Nevertheless, the routine laboratory tests can only analyze factors in plasma and isolated components or fractions. Thus, it does not adequately evaluate the whole coagulation state in patients with ATAAD. At many major cardiovascular centers, TEG-guided perioperative bleeding management has been extensively used to monitor hemostasis and decrease the risk of bleeding (34, 35). This technology may provide an overall view of coagulation and detect platelet function (MA level) and fibrinogen function (αangle), which may be beneficial for patients with ATAAD. Although TEG has been proven to reduce transfusions in cardiac surgery, only a few studies have shown the predictive value of TEG (11, 36, 37). Nevertheless, the use of TEG as a tool to predict risk factors for severe postoperative AKI (stage 3) has been confirmed by our study. TEG measurements could supplement routine laboratory tests, but they do not negate the need for routine laboratory tests.

### Study limitations

Several potential limitations of the present study should be discussed. First, the sample size of the study was small, and this study was conducted only in one institution, which may limit the applicability of our findings to other settings. Second, we could not identify the underlying mechanisms linking the hemostatic system to the development of ATAAD-AKI. Third, some potential bias may have been retained after the multivariate analysis. To verify these findings, larger and more comprehensive prospective multicenter studies are needed. Finally, further long-term follow-up studies are needed to better understand the association between the preoperative hemostatic system and postoperative ATAAD-AKI.

## Conclusions

In conclusion, in the present study, we found that preoperative fibrinogen level and MA level were significantly associated with the risk of severe postoperative AKI (stage 3) in patients with ATAAD. The TEG may be an effective tool in identifying and assessing the risk factors for severe postoperative ATAAD-AKI (stage 3) in patients with ATAAD (stage 3).

## Data availability statement

The original contributions presented in the study are included in the article/supplementary material, further inquiries can be directed to the corresponding authors.

## Ethics statement

The studies involving human participants were reviewed and approved by the Ethics Committee at Anzhen Hospital (Institutional Review Board File No. 2018004). The patients/participants provided their written informed consent to participate in this study. Written informed consent was obtained from the individual(s) for the publication of any potentially identifiable images or data included in this article.

## Author contributions

Conception and design: X-LG, H-YL, H-JZ, and X-LW. Administrative support: H-YL and H-JZ. Provision of study materials or patients: MG and X-LW. Collection and assembly of data: X-LG, MG, and H-YL. Data analysis and interpretation: X-LG, LL, and X-LW. All authors contributed to the article and approved the submitted version.
